# Effects of a mannan-rich yeast cell wall-derived preparation on cecal concentrations and tissue prevalence of *Salmonella* Enteritidis in layer chickens

**DOI:** 10.1371/journal.pone.0232088

**Published:** 2020-04-23

**Authors:** G. Girgis, M. Powell, M. Youssef, D. E. Graugnard, W. D. King, K. A. Dawson

**Affiliations:** 1 Nevysta Laboratory, Iowa State University Research Park, Ames, IA, United States of America; 2 Alltech Center for Animal Nutrigenomics and Applied Animal Nutrition, Nicholasville, KY, United States of America; USDA-Agricultural Research Service, UNITED STATES

## Abstract

*Salmonella* Enteritidis (SE) has been the most common *Salmonella* serotype associated with foodborne infections in the last several years. Dietary applications of yeast-based preparations in feed have shown to reduce *Salmonella* colonization in chickens augmenting SE control strategies. This study was conducted to evaluate the effects of a mannan-rich yeast cell wall-derived preparation (Actigen^®^) administered in feed at a rate of 400 g/ton on SE colonization in the cecum and internal organs of commercial layer chickens. Sixteen week-old layer pullets were orally challenged with a selected nalidixic acid resistant SE strain at a dose of 1.7×10^9 colony forming units (CFU) per bird. SE colonization was assessed by evaluating isolation rates from ovary and pooled liver/spleen samples as well as enumeration of SE in cecal pouches one week post-challenge. Recovery rates of SE from the ovaries of directly challenged birds receiving Actigen^®^ were significantly lower (*P* <0.02) than those in directly challenged birds fed an unsupplemented control diet. Recovery rates of SE from pooled liver/spleen samples were not significantly different between Actigen^®^-treated pullets and controls (*P* = 0.22). Using direct plate count methods, cecal SE concentrations were 1 log_10_ lower (*P* <0.001) in challenged pullets in the Actigen^®^-supplemented group than in the challenged controls. The SE concentration distributions in the ceca were similar in groups testing positive and groups testing negative for SE in the ovaries and liver/spleens tissues. As a result, SE concentrations in the ceca could not be directly related to the occurrence or prevalence of SE in these tissues. In conclusion, Actigen^®^ supplementation appears to decrease the prevalence of SE in ovarian tissue and concentrations of SE in cecal contents and may be useful as a tool for reducing the risk of eggshell contamination and transovarian transmission of SE in eggs.

## Introduction

A recent report of the Foodborne Diseases Active Surveillance Network has stated that *Salmonella* continues to be the second most common infection in the United States, and that *Salmonella* Enteritidis (SE) is the most common serotype [1]. SE is adapted to live in poultry, and eggs are an important source of infection [2]. The incidence of human infections with SE has not declined in over 10 years [1, 3]. The percentage of samples of chicken intestinal contents that yielded SE in the United States were similar in 2018 to those during 2015–2017 [3]. Some decline in serotype Typhimurium observed during the same period was thought to be associated with the use of modified live *Salmonella* vaccines, which are made of *Salmonella* Typhimurium [4]. In the United Kingdom, the use of modified live SE auxotrophic vaccine was followed by a decrease in human SE infections [5]. However, even with the available modified live vaccines, *Salmonella* infections still represent a major problem in the United States. Other strategies that reduce colonization of layers and breeders are needed to augment available vaccines and management practices to achieve further reductions of *Salmonella* in eggs and poultry meat [6].

On the other hand, the emergence of multidrug-resistant pathogens and concerns about the transference of such antibiotic resistance to human microbiota led to restrictions on the use of many antimicrobials that are used as the base for pathogen control programs in the poultry industry [7, 8]. Therefore, the focus has shifted to developing and exploring alternative feed additives that help retain poultry performance, improve health and reduce the prevalence of foodborne pathogens, particularly *Salmonella* [9].

Feed application of mannan oligosaccharides (MOS) derived from yeast cell wall have been shown to have bacterial binding characteristics [10] and agglutinate different species of *Salmonella* in laboratory tests. It is believed that these characteristics contribute to reduced colonization of *Salmonella* in the gastrointestinal tract of chickens [11, 12]. Additionally, mannan-rich yeast preparations have been shown to initiate immune responses by macrophages and dendritic antigen presenting cells [13] and to beneficially modify the composition of the gastrointestinal microbiome [14]. The objective of this study was to evaluate the effects of a mannan-rich yeast cell wall preparation, Actigen®, on SE colonization in the cecum and internal organs of commercial layer pullets.

## Materials and methods

### Experimental animals

One hundred and eighty four one-day-old W-36 commercial layer pullets were obtained from Hy-Line North America, LLC (Warren, IN). Pullets received infrared beak treatment and were vaccinated against Marek’s disease at the hatchery. Pullets were reared in two-tiered, wire mech, pullet cages measuring 24”x20”x15” at a stock rate of 16 birds per cage until 6 weeks of age at which point stocking density were reduced to 8 birds per cage. Temperature and lighting strategies were provided as specified in the Hy-line W-36 pullet manual. Pullets were housed and received the SE challenges at the AAALAC-accredited Laboratory Animal Resources (LAR) isolation facility of Iowa State University (ISU). Birds were managed as has been prescribed by the Guide for the Care and Use of Agricultural Animals in Research and Teaching [15] with the study protocol approved by the Institutional Animal Care and Use Committee (IACUC) and the Institutional Biosafety Committee (IBC) of ISU. The behavior and general health condition of the pullets were observed daily.

### Experimental diets

A corn- and soybean meal-based non-medicated mash control diet appropriate to the age was formulated to meet the standard nutritional specifications for layer pullet chickens [16]. Diet supplemented with yeast cell wall preparation contained 400 gm of Actigen® (Alltech, Inc., Nicholasville, KY) per ton. No chemical or microbiological contaminants that may interfere with the study were known to be present in experimental diets. Routine bacteriological analysis was conducted on each batch of the feed used in the study to confirm absence of *Salmonella* contamination. The concentration of the yeast preparation in the feed was confirmed to be 0.39±0.03 kg/ton using an enzyme-linked mucin adherence assay.

### Experimental design

One-day-old pullets were randomly divided into two groups (92 pullets/group), one group was fed control diets and the other group was fed Actigen®-supplemented diets. Birds were fed appropriate diets *ad libitum* from day 1 till the end of study at 17 weeks of age. Appropriate temperature, humidity, and lighting programs were followed according to the recommendations of the Hy-Line W-36 manual. At the age of 12 weeks, pullets were moved to 3-tier cage units (30ʺ×30ʺ×18ʺ) for acclimatization. Group housing, 4 pullets per cage, was used with each cage having wire-mesh floor and manure collection tray underneath in order to reduce fecal oral re-infection after challenge (collection trays were cleaned daily). At this point, duration of lighting periods was gradually increased from 12 hours to 16 hours to stimulate ovarian development and simulate stress associated with onset of egg production. Light duration reached 16 hours prior to administration of challenge doses through the end of the study. At the age of 16 weeks, two pullets in each cage (*n* = 46 per group) were directly challenged with an oral dose of a selected nalidixic acid resistant strain of SE at the concentration of 1.7×10^9 CFU. The two remaining birds in each cage did not directly receive a SE challenge but were maintained in the cage with directed challenged birds to evaluate contact challenge. Pullets were humanely euthanized by cervical dislocation and sampled 7-days post-challenge.

### Sample collection and processing

At 15 weeks of age (prior to challenge), drag swabs were collected from the manure trays underneath each cage to confirm the absence of SE prior to challenge. Each swab was placed in sterile sampling bag and gloves were changed between samples. At 17 weeks of age (7 days after direct challenge), all birds were humanly euthanized and liver, spleen, ovary and ceca pouches were aseptically collected from individual birds and placed in separate sterile sampling bags. Bags were kept on ice packs and transported to the laboratory for immediate analyses. Drag swabs were also collected from individual manure trays underneath cages at 17 week of age and tested for the presence of SE. Each swab was placed in sterile sampling bag and gloves were changed between samples.

### *Salmonella* isolation and identification

Drag swabs were processed for *Salmonella* isolation and identification using pre-enrichment in buffered peptone water for 24 hours at 37°C followed by enrichment in Tetrathionate Hajna (TTH) broth for 18–20 hours at 42°C. Plating was done on Xylose Lysine Tergitol-4 (XLT-4) and Brilliant Green agar. Any suspected colonies were further tested in Triple Sugar Iron (TSI) and Lysine Iron (LIA) slants followed by serogrouping using appropriate O and H *Salmonella* antisera.

Ovary and pooled liver/spleen samples from each bird were separately added to TTH broth at a ratio of 1:10 weight to volume. Samples were mixed using stomacher then incubated at 42°C for 24 hours. Incubated media were streaked on XLT-4 agar plates containing 25μg of nalidixic acid/mL. Suspected colonies were further tested in TSI and LIA slants followed by serogrouping using appropriate O and H *Salmonella* antisera to confirm the inoculum strain.

### Enumeration of *Salmonella* in cecal pouches

Enumeration of the challenge SE strain in cecal contents was done by direct plate count method [17] using saline as diluent and XLT-4 agar plates containing 25μg of nalidixic acid/mL. Agar plates were incubated for 24 hours at 37°C and typical *Salmonella* colonies were counted within suitable colony counting range of 25–250. At least three randomly selected colonies from each positive sample were serologically confirmed to be the SE inoculum strain to validate the accuracy of the visual counts.

Challenge SE strain was also enumerated using the miniatured Most Probable Number (MPN) method described earlier [6, 18]. Briefly, 1 mL of sample was transferred to 3 adjacent wells in the first row of a 96-well 2 mL deep well plate and 0.1 mL aliquot was transferred to 0.9 mL of TTH broth with nalidixic acid in the second row. This process was repeated for remaining rows producing five 10-fold dilutions. Plates were incubated for 24 hours at 42°C. One microliter of each well was transferred onto XLT-4 agar containing nalidixic acid and incubated for 24 hours at 37°C. SE inoculum strain was confirmed serologically using appropriate O and H antisera.

### Statistical analysis

SE recovery rates and *Salmonella* prevalence in tissues were compared using a Fisher’s exact test. SE concentration were subject to log_10_ transformation prior to evaluation. Log SE concentration distributions in pullets and dot blot evaluations were examined using the data Distribution and Graph Builder procedures in the JMP software package from SAS (JMP, SAS Institute Inc., Cary, NC).

## Results

### *Salmonella* culture from environmental swabs

All environmental swabs collected pre-challenge tested negative for SE. All drag swabs collected from manure trays 7 days post-challenge were positive for SE, confirming the successful establishment of SE infection in directly inoculated birds.

### Prevalence of *Salmonella* in tissues

Overall, *Salmonella* was detected predominantly in the ceca and combine liver and spleen of directly challenged birds compared to the contact challenge birds regardless of the dietary treatment. In the ovary tissue, the direct challenge birds also presented greater percentages of positive *Salmonella* compared to the contact challenged birds. However, the relative number of directly challenged birds testing positive for *Salmonella* in ovary tissue was significantly lower (*P <* 0.02) in birds receiving the Actigen®-supplemented diet compared to controls (Table 1).

**Table 1:** Effects of a mannan-rich yeast cell wall dietary supplement on the detected frequency of nalidixic acid resistant *Salmonella* Enteritidis in the tissue samples from 17-week-old layers 1 week after direct or contact challenge.

	Actigen®		Control	
Tissue	Direct Challenge	Contact Challenge	Direct Challenge	Contact Challenge
Cecum	46/46 (100%)^a^	15/46 (32.6%)^b^	46/46 (100%)^a^	16/46 (34.8%)^b^
Ovary	13/46 (28.3%)^b^	2/46 (4.3%)^c^	23/46 (50%)^a^	3/46 (6.5%)^c^
Liver and Spleen	43/46 (93.4%)^a^	10/46 (21.7%)^b^	41/46 (89.1%)^a^	12/46 (26.1%)^b^

Values represent the number of *Salmonella* positive birds/total number of birds tested. Numbers in parenthesis represent the percentage of positive birds. Values with different superscripts within a row are significantly different (*P* < 0.02).

### *Salmonella* concentrations in ceca

*Salmonella* concentrations analyzed by the MPN method in both experimental groups were greater than the 10^5^ CFU/g upper limits of the assay. These measures were considered unreliable for treatment comparisons and were not used for any statistical calculations.

**Fig 1:**
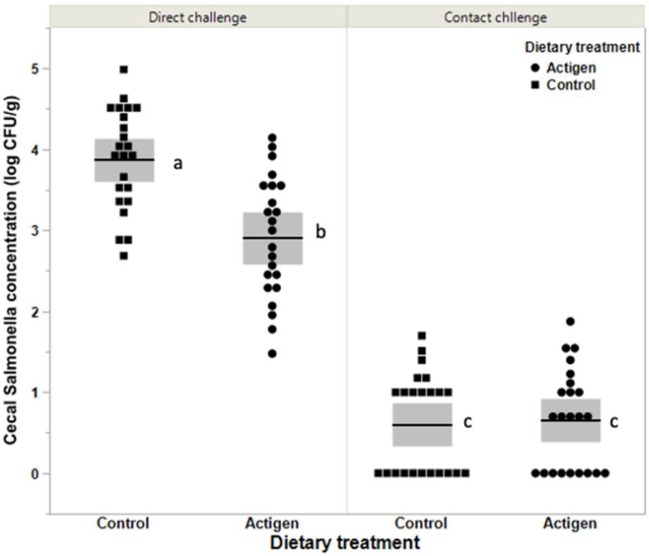
Box plot of the mean concentrations (log_10_ CFU/g) of *Salmonella* in cecal contents from 17 week-old layers 1 week after a direct or contact challenge with *S*. Enteritidis as influenced by dietary treatment (46/treatment). The boxes represent mean and standard deviation values. Birds were fed either basal diet (Control) or a diet supplemented with 400 g of Actigen®/ton from day of hatch through week 17. Direct challenged birds were challenge on week 16 with 1.7 X 10^9^ CFU of nalidixic acid resistant *S*. Enteritidis. Contact challenged birds were exposed to *S*. Enteritidis by housing them in the same cage as the direct challenged birds. The mean concentrations (log_10_ CFU/g) of *Salmonella* in the ceca of the direct challenged birds were 2.9±0.8 and 3.9±0.6 for the Actigen® and Control groups, respectively. The mean concentration of salmonella in the ceca from contact challenged birds were 0.6±0.6 and 0.7±0.6 for the Actigen® and control groups, respectively. Means designated by different letters (a, b and c) are significantly different (*P* < 0.001).

The average cecal *Salmonella* concentration (log_10_ CFU/g) in each cage evaluated by direct plate count method in pullets fed the Actigen®-supplemented diet was significantly lower (*P <* 0.001) than that observed in the control (Fig 1). Overall, the average cecal SE concentration in the Actigen®-supplemented group (2.9±0.8) was one log_10_ lower than that in the control group (3.9± 0.6). The concentration of *Salmonella* in the ceca from contact-challenged birds Actigen®-supplemented (0.7±0.6) or controls (0.6±0.6) were not influenced (*P =* 0.79) by the dietary treatment. However, the average of *Salmonella* concentrations (0.6±0.6) in the ceca of birds subject to the contact challenge was significantly lower (*P <* 0.001) than those observed in the birds that were subject to the direct challenge (3.4±0.7).

### *Salmonella* concentration distributions and tissue colonization in direct-challenged birds

**Fig 2:**
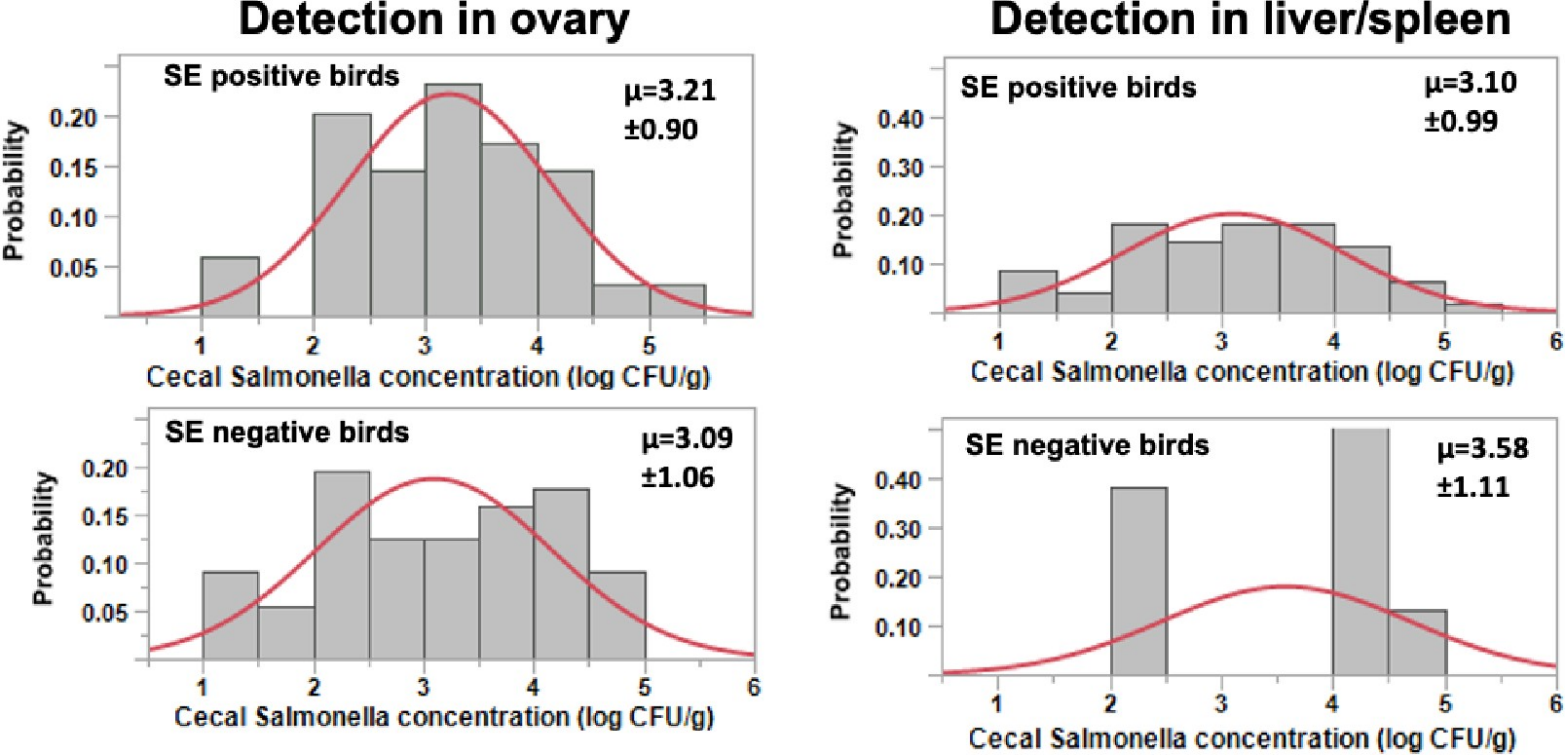
Cecal *Salmonella* concentration (log_10_ CFU/g) distributions in *Salmonella* positive and negative samples from the ovary and liver/spleen of 17 week-old layers 1 week after direct challenge with *S*. Enteritidis. The bar graphs show the frequency of each concentration range while the red line shows the normal distribution curve based on the observed concentration. Detection was based on the occurrence of *Salmonella* in the tissue as determined by growth after plating on XLT-4 agar plates containing 25μg of nalidixic acid/mL. μ represents the mean log_10_ concentration ± the standard deviation.

Regardless of the dietary treatments, the distribution and mean of cecal *Salmonella* concentrations were similar in groups of birds with and without detectable levels of *Salmonella* in the ovary and the pooled liver/spleen (Fig 2). Interestingly, a significant number (>50%) of the birds that tested negative for *Salmonella* in the ovary and the pooled liver/spleen had cecal *Salmonella* concentrations with over 3 and 4 log_10_ CFU/g, respectively. In contrast, a significant number (>50%) of the birds that tested positive for *Salmonella* in the evaluated tissues had cecal *Salmonella* concentrations under 3 log_10_ CFU/g. The colonization of the evaluated tissues did not follow a cecal *Salmonella* concentration dependent response.

## Discussion

Enumeration of *Salmonella* following an experimental challenge has routinely been used as an indication of potential shedding and the potential for foodborne illness [19]. In this study, the dietary inclusion of a mannan-rich yeast cell wall-derived preparation (Actigen®) significantly decreased colonization rates of SE in the ceca of challenged birds as determined by direct plate count method. This observation is consistent with earlier studies demonstrating that yeast products helped in reducing *Salmonella* colonization in the intestinal tract of chickens [10–12, 20–22]. The lower *Salmonella* colonization rates detected in this study suggests a potential beneficial impact of Actigen® on eggshell contamination with *Salmonella* and subsequently public health.

The establishment of *Salmonella* infection may involve a subtle interplay between host susceptibility and the dose of a specific challenge strain of SE [23]. Susceptibility to intestinal colonization and organ invasion have been reported to vary between lines of chickens as well as housing systems [24, 25]. High isolation rates from cecum, liver and spleen reported in this study are in agreement with previous studies [23]. SE recovery rates in ovaries were significantly different between challenged birds in the Actigen®-supplemented group and controls (*P <* 0.02). While colonization as measured by *Salmonella* concentrations in cecal contents is often used to evaluate *Salmonella* mitigation strategies, colonization of reproductive tissue remains to be the pivotal step in the sequence of events that leads to the deposition of SE in eggs [25, 26]. Colonization of reproductive tissues and production of contaminated eggs is critical to the usefulness of experimental infection models for developing and evaluating SE control strategies [25]. Reduction of SE colonization in the ovary of birds receiving Actigen® supplemented feed highlights its potential value for controlling *Salmonella* in eggs.

In this study, there does not appear to be a clear relationship between the concentration of *Salmonella* in the cecum and the rates of colonization in the ovaries or liver/spleen tissue. Mean, distribution and range of cecal *Salmonella* concentrations were similar for birds testing positive for *Salmonella* and in those with no detectable *Salmonella* in both ovarian and liver/spleen tissues. Moreover, most of the birds testing negative for *Salmonella* in the ovary and/or in the combine liver and spleen tissue had significant levels of *Salmonella* in cecal contents. These observations suggest that measures of *Salmonella* concentrations in cecal contents by itself does not reflect the potential for *Salmonella* colonization in other tissues and the possibility for transovarian transmission of the pathogen.

Acute challenge experiments often use miniatured MPN technique that are less laborious than direct plate count methods. However, for high pathogen concentrations such as those derived during experimental challenges, the values obtained by MPN may not necessarily be as accurate as those values derived from direct plating methods. MPN values are only estimates based on probability theory applied to matrices having low numbers (<100/g) of *Salmonella* [27]. This may explain the variations between obtaining enumerable plate count values while the MPN upper limit was saturated. Adjusting over-the-limit MPN readings to a single value suggested elsewhere [6] was not considered in this study as they do not represent true counts appropriate for statistical analysis. The current study confirmed the usefulness of the direct plate count method as a quantitative tool for estimating concentration of a marked SE challenge strain. In addition to the appropriate methods for evaluation, further research at lower direct challenge numbers is warranted to more accurately reflect environmental dosages, and some analysis of strain variation should be explored also [28, 29].

In conclusion, our study confirmed that the supplementation of Actigen® significantly reduced both the prevalence of *Salmonella* in the ovary tissue and the concentration of *Salmonella* in the ceca of layer pullets after an immediate SE challenge. The dietary administration of Actigen® may be useful in strategies for reducing the risk of eggshell contamination and transovarian transmission in egg production systems.

## Supporting information

S1 File Raw data of *Salmonella* positives and negatives in ovary and combined liver/spleen and cecal *Salmonella* concentration (CFU/g) of 17 week-old layers 1 week after direct challenge with *S*. Enteritidis. (XLSX)
